# Human Multi-Chimeric Cell (HMCC) Therapy as a Novel Approach for Tolerance Induction in Transplantation

**DOI:** 10.1007/s12015-023-10608-z

**Published:** 2023-08-21

**Authors:** Maria Siemionow, Joanna Cwykiel, Sonia Brodowska, Lucile Chambily

**Affiliations:** 1 Department of Traumatology, Orthopaedics and Hand Surgery, Poznan University of Medical Sciences, Poznan, Poland; 2 Department of Orthopaedics, University of Illinois at Chicago, 900 South Ashland Ave., Room# 3356, Molecular Biology Research Building, Chicago, IL 60607 USA

**Keywords:** Cellular therapy, Chimerism, Umbilical cord blood (UCB), Human multi-chimeric cell (HMCC) therapy, Tolerance induction, Vascularized composite allotransplantation (VCA)

## Abstract

Cellular therapies are regarded as the most promising approach for inducing transplant tolerance without life-long immunosuppression in solid organ and vascularized composite allotransplantation (VCA). Currently, no therapies are achieving this goal. This study introduces a novel Human Multi-Chimeric Cell (HMCC) line created by fusion of umbilical cord blood (UCB) cells, from three unrelated donors as an alternative therapeutic approach to bone marrow transplantation and tolerance induction in solid organ and VCA transplants. We performed eighteen ex vivo polyethylene glycol mediated fusions of human UCB cells from three unrelated donors to create HMCC. Mononuclear cells labeled with PKH26, PKH67, and eFluor™ 670 fluorescent dyes were fused and sorted creating a new population of triple-labeled (PKH26/PKH67/eFluor™ 670) HMCC. The creation of HMCC from three unrelated human UCB donors was confirmed by flow cytometry and confocal microscopy. Genotyping analyses determined the tri-chimeric state of HMCC by presence of parent alleles and selected loci specific for each of three UCB donors. Phenotype characterization confirmed hematopoietic markers distribution, comparable to UCB donors. HMCC maintained viability and displayed a low apoptosis level. The COMET assay revealed absence of genotoxicity, confirming fusion safety. Colony forming units assay showed clonogenic properties of HMCC. This study confirmed the feasibility of HMCC creation from three unrelated human UCB donors and characterized tri-chimeric state, hematopoietic phenotype, viability, safety, and clonogenic properties of HMCC. The created HMCC line, representing genotype characteristics of three unrelated human UCB donors, introduces a novel therapeutic approach for bone marrow, solid organ, and VCA transplants.

**Graphical Abstract:**

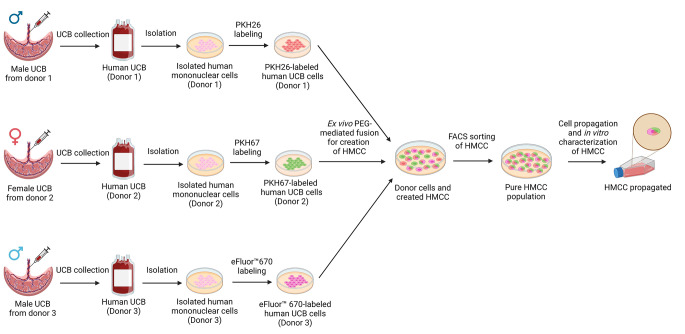

**Supplementary Information:**

The online version contains supplementary material available at 10.1007/s12015-023-10608-z.

## Introduction

Over 41,000 transplants of solid organs and bone marrow are performed annually in the United States. With the numbers increasing each year, the demand for new immunomodulatory treatments is on rise [1]. In recent decades, vascularized composite allotransplantation (VCA) including, face, hand, larynx, and uterus transplants, was introduced as a new field into the armamentarium of transplantation and revolutionized both, the transplant medicine and reconstructive surgery. Due to the distinct immunogenic responses triggered by different transplant components, a major concern in transplant surgery revolves around the necessity for lifelong multidrug immunosuppression (IS) to counter transplant rejection. However, the significant side effects of IS hinder the routine clinical application of VCA [2, 3]. In the past decade, the FDA has not approved any new tolerance-inducing protocols for solid organ transplants (SOT), bone marrow transplants (BMT), or VCA that would enable these transplantation procedures without IS [4, 5]. Hence, the paramount objective of transplantation research is to attain transplant tolerance with minimal or no reliance on IS.

Recently, there has been increasing interest in cellular therapies aimed at suppressing or modulating the immune response following SOT, BMT, and VCA, either by reducing or eliminating the lifelong IS [6, 7]. At present, one of the approaches for tolerance induction in VCA involves co-transplantation of donor-origin bone marrow (BM). BM-based cellular therapy has undergone clinical testing in scenarios involving SOT and VCA, such as kidney and upper-extremity transplantation, respectively [8–10]. However, the combined IS and BM-based cellular protocols lead to numerous side effects including graft-versus-host disease (GVHD) [11]. Hence, therapies centered around modulating the recipient’s immune response and fostering tolerance, offer the potential to reduce or eliminate the need for IS.

In previous reports, we introduced a novel therapy involving ex vivo created donor-recipient chimeric cells (DRCC) as an alternative approach to BM-based cellular therapies, aiming to support tolerance induction in VCA [6, 12, 13]. The successful development of rodent DRCC introduced the concept of mixed chimerism and the methodology for generating human chimeric cells via the ex vivo PEG-mediated fusion procedure. This concept of universal chimeric cell therapy for tolerance induction in transplantation garnered significant interest and support within the transplant community [14, 15].

Building upon the promising preclinical data, we proceeded to develop, characterize, and verify the in vitro the safety of a new cellular therapy involving human hematopoietic chimeric cells (HHCC). These cells were created from CD34^+^ cells derived from human BM through the ex vivo PEG-mediated fusion procedure [16]. With the intention of progressing this innovative concept of cell-based therapy into clinical trials, our objective was to establish and characterize a universal chimeric cell therapy as an “off-the-shelf” product designed to enhance allograft survival in SOT, BMT, and VCA. To realize this objective, we opted to utilize umbilical cord blood (UCB) cells as an alternative reservoir of hematopoietic stem cells aiming to induce tolerance in transplantation and regenerative medicine contexts. UCB cells have garnered attention as a potential alternative treatment for autoimmune, infectious, and immunodeficiency conditions across the pediatric and adult population [17]. The heightened accessibility and limited immunogenicity render UCB a compelling candidate for the establishment of a novel hematopoietic cell line.

Our earlier investigation which introduced the formation of HHCC laid the groundwork for the development of an innovative UCB-based hematopoietic cell line of the human umbilical di-chimeric (HUDC) cells [18]. The HUDC study substantiated evidence for a closer HLA-match between the human donor and the recipient, achieved through the fusion of human UCB cells from two unrelated donors. In the present study, the objective was to attain a closer donor-recipient HLA-match by fusing human UCB cells from three unrelated donors. This endeavor aimed to establish the subsequent generation of human multi-chimeric cells (HMCC)-based therapy. This study successfully confirmed the feasibility, reproducibility, and safety associated with the creation of HMCC via ex vivo PEG-mediated fusion of UCB cells derived from three unrelated human donors. Furthermore, we verified the genotype, hematopoietic phenotype, viability, safety, and clonogenic properties of HMCC. Consequently, this study introduces an innovative cell-based therapy tailored for tolerance induction in transplantation.

## Materials and Methods

### Creation of Novel Human Multi-Chimeric Cell (HMCC) Line

#### Cells Isolation from Three Unrelated UCB Donors

In this study, the human UCB units were purchased from the Cleveland Cord Blood Bank. The UIC Office for the Protection of Research Subjects has determined that this activity does not meet the definition of human subjects’ research as defined by the 45 Code of Federal Regulations (CFR) 46.102(f). No ethical approval or informed consent was required due to the nature of this study. Density gradient centrifugation (Lymphoprep™, StemCell™ Technologies, Vancouver, Canada) was used to isolate UCB cells from three unrelated human UCB donors. Next, the UCB samples were centrifuged for 25 min at 300 g, and collection of cells in interphase was performed. The UCB cell purification was performed with the anti-human CD235a (Glycophorin A) MicroBeads and magnetic-activated cell sorting (MACS) (MACS^®^, Miltenyi Biotec, Bergisch Gladbach, Germany), according to the manufacturer’s instructions. The isolated UCB cells were washed in RPMI 1640 medium containing 10% FBS (Thermo Fisher Scientific) and 1X antibiotic/antimycotic solution, then suspended for further analysis.

### PEG-mediated Cell Fusion Procedure

**Fig. 1:**
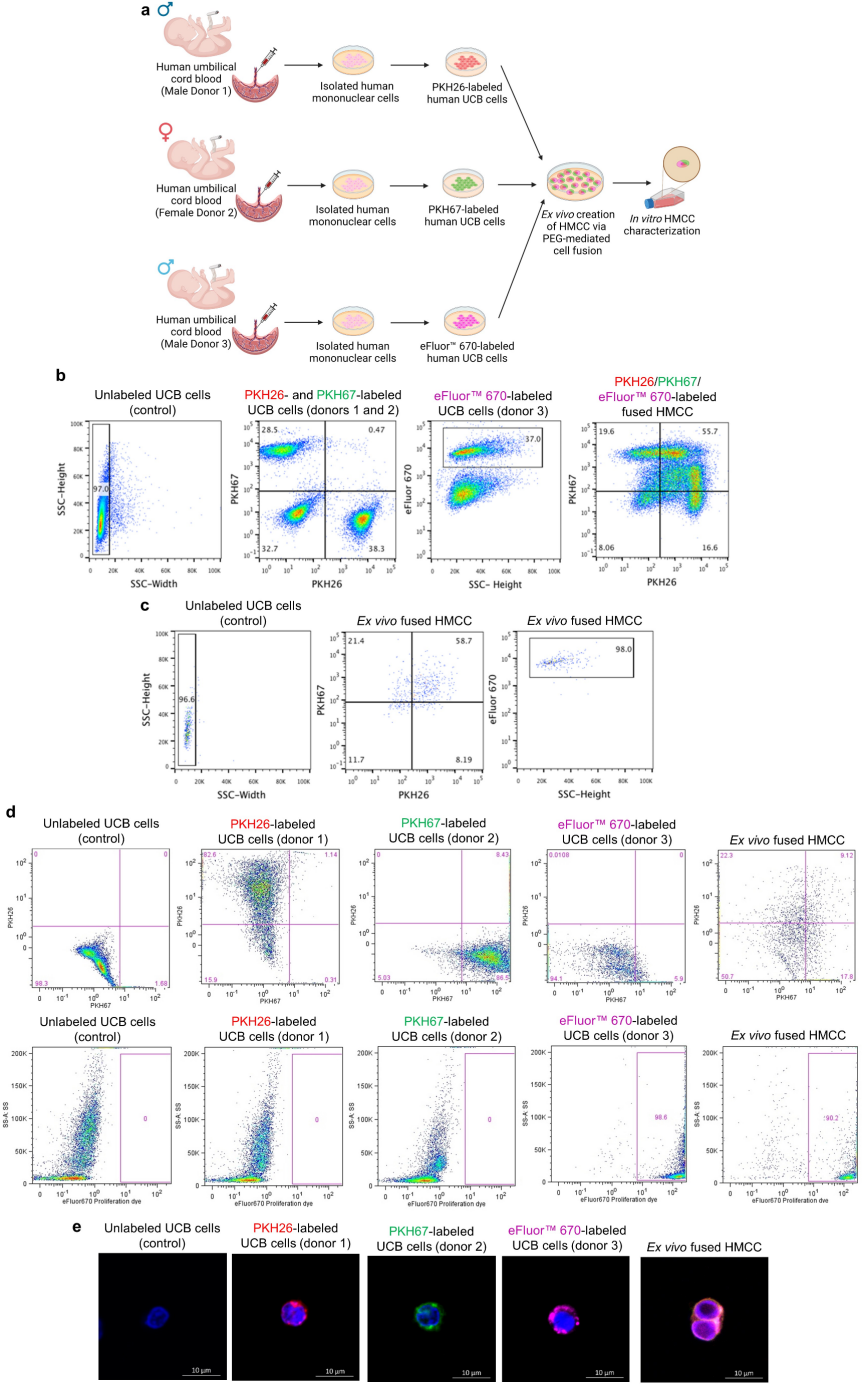
Confirmation of creation of the Human Multi-Chimeric Cells (HMCC) line from three unrelated UCB donors via ex vivo PEG-mediated fusion procedure. (**a**) The study design of ex vivo polyethylene (PEG)-mediated cell fusion procedure of creating a novel HMCC line from three unrelated donors (two males and one female) of umbilical cord blood (UCB) cells. (**b-e**) Confirmation of efficacy of HMCC creation via ex vivo fusion procedure and assessment of HMCC population after cell sorting: (**b**) Representative fluorescence-activated cell sorting (FACS) dot-plots presenting gating strategy for HMCC sorting after fusion procedure (in sequential order from left): SSC-height vs. SSC-width plot for aggregate correction, mixed PKH26- and PKH67-labeled UCB control cells gated on PKH67- vs. PKH26-labeling; eFluor™ 670-labeled UCB control cells gated based on eFluor™ 670-labeling vs. SSC-height.; and ex vivo fused PKH26/PKH67/eFluor™ 670-labeled HMCC gated based on PKH67- vs. PKH26-labeling; (**c**) Representative flow cytometry (FC) dot-plots presenting fluorescent dyes for confirmation of labeling efficacy of UCB control cells before fusion and HMCC after sorting (in sequential order from left): unlabeled; PKH26-; PKH67-; and eFluor™ 670-labeled UCB control cells; and ex vivo fused PKH26/PKH67/eFluor™ 670-labeled HMCC gated based on upper row panels: PKH26- vs. PKH67-labeling and lower row panels: SS-A vs. eFluor™ 670-labeling; (**d**) Representative FC dot-plots presenting the purity of HMCC population after sorting (in sequential order from left): SSC-height vs. SSC-width plot for aggregate correction, ex vivo fused PKH26/PKH67/eFluor™ 670-labeled HMCC gated based on PKH67- vs. PKH26-labeling, and eFluor™ 670-labeling vs. SSC-height. (**e**) Representative immunofluorescence confocal microscopy (CM) images of (in sequential order from the left): unlabeled human UCB cells (control); PKH26-labeled human UCB cells of donor 1 (red); PKH67-labeled human UCB cells of donor 2 (green); eFluor™ 670-labeled UCB cells of donor 3 (purple); and the ex vivo fused HMCC, revealing the overlapping of PKH26/PKH67/eFluor™ 670 fluorescent dyes, confirming the tri-chimeric state of the created HMCC. Images were captured using a upright confocal microscope (Leica TCS SP2 Upright Confocal Microscope), scale bar: 10 μm

The HMCC were created from UCB cells derived from three unrelated human donors as presented in Fig. 1a. The isolated UCB donor cells were fluorescently labeled using traceable dyes: PKH26 - red, PKH67 - green (MiliporeSigma, Burlington, MA, USA), and eBioscience™ Cell Proliferation Dye eFluor™ 670 - purple (Thermo Fisher Scientific, Waltham, MA, USA) according to the manufacturer’s instructions. The cells of each donor labeled separately with either PKH26, PKH67, or eFluor™ 670 dye were mixed in the ratio of 1:1:1 and washed with serum-free RPMI 1640 medium (Thermo Fisher Scientific). The cell count was measured manually using the Burker counting chamber (Graticules Optics, Kent, UK) and automatically during the sorting process to obtain the appropriate starting cell count for each UCB donor to perform the cell fusion procedure. From each UCB donor, 2 × 10^7^ cells were counted for the first fusion and 1 × 10^7^ cells for the following fusions. The cell fusion procedure was performed using polyethylene glycol (PEG) 4000 solution (EMD, Burlington, MA, USA), as previously reported [13, 16, 19, 20]. The fluorescence-activated cell sorting (FACS, BD FACSAria^™^ II cell sorter, Becton Dickinson, Franklin Lakes, NJ, USA) was applied to select the PKH26/PKH67/eFluor™ 670-labeled cells, representing the HMCC population. A total of 18 fusions were performed. The unlabeled UCB and single labeled UCB control cells were used to optimize the sorter settings. The gating strategy for sorting the HMCC population is presented on Fig. 1b. Immediately after completion of sorting, HMCC were re-analyzed using the established sorting setting and gating strategy to confirm the purity of the sorted HMCC population.

### Verification of HMCC Creation by Flow Cytometry and Confocal Microscopy

Confirmation of HMCC creation via ex vivo PEG-mediated cell fusion procedure was assessed by flow cytometry (FC) and confocal microscopy (CM). Assessment of staining efficacy and verification of HMCC creation was evaluated by FC. In addition, FC assessed fusion procedure efficacy (n = 5). To confirm HMCC creation by CM, samples of the isolated unlabeled UCB, PKH26-, PKH67-, and eFluor™ 670-labeled UCB control cells, and HMCC were spun onto Fisherbrand™ Superfrost™ Plus Microscope Slides (Fisher Scientific, Waltham, MA, USA), fixed in 4% paraformaldehyde (EMS, Hatfield, PA, USA) for 15 min at room temperature, and mounted with VECTASHIELD^®^ Antifade Mounting Medium with DAPI (Vector Laboratories, Burlingame, CA, USA). The slides were assessed using an upright confocal microscope (Leica TCS SP2 Upright Confocal Microscope, RRID:SCR_020231, Leica Microsystems, Wetzlar, Germany) with a digital camera (QImaging^®^ Retiga-2000R Charge Coupled Device, QImaging, British Columbia, Canada) and ImagePro Plus (RRID:SCR_016879, Media Cybernetics, Rockville, MD, USA) software.

### Assessment of HMCC Genotype by PCR-rSSOP and PCR-STR

The DNA samples of the UCB donor cells and the created HMCC were typed using PCR-rSSOP method for detection of human leukocyte antigens (HLA)-A, -B, -C, -Bw, -DRB1, -DQB1, and -DR51/DR52/DR53 using commercial kits (LABtype rSSO Typing Test, Thermo Fisher Scientific). According to the manufacturer’s instructions, the DNA isolation was performed with the DNeasy Blood and Tissue Isolation kit (Qiagen, Hilden, Germany). The DNA samples were subjected to PCR amplification (PE9700 Thermo Cycler Life Technologies, Carlsbad, CA, USA) as previously reported [16]. Next, StreptAvidin PhycoErythrin conjugate (Thermo Fisher Scientific) was added to the products for 5 min at 60 °C. The products were then suspended with 60 μL of washing buffer, fluorescence signals were analyzed using the laser Luminex^®^ 200^™^ system (Luminex, Austin, TX, USA), and HLA typing was obtained by the Tissue Typing Laboratory (University of Illinois Hospital and Health Sciences System, Chicago, USA).

The presence of short tandem repeat (STR) loci specific for each of the three fusion donors in the HMCC population was determined by Short Tandem Repeat-Polymerase Chain Reaction (STR-PCR) analysis after completion of the fusion procedure. Genetic fingerprinting was performed using Promega’s GenePrint 10 kit (Promega Corporation, Madison, WI, USA) according to the manufacturer’s protocol. The DNA was extracted from three donors and HMCC cell pellets using the Maxwell^®^ 16 Tissue DNA purification kit (Promega Corporation). One pellet per sample was transferred to the cartridge, and the cell extraction protocol was run according to the manufacturer’s instructions. Then, the extracted DNA was amplified using Biosystems ABI 3730 DNA Analyzer (Thermo Fisher Scientific) and AmpFLSTR^™^ Identifiler^™^ PCR Amplification kit (Thermo Fisher Scientific). The STR-PCR analysis was conducted as previously reported [16]. Appropriate positive and negative controls were prepared alongside the samples. Data were obtained for the following genetic markers (STR loci): TH01, D21S11, D5S818, D13S317, D7S820, D16S539, vWA, and TPOX, and then uploaded to the GeneMapper^™^ 5.0 analysis software (RRID:SCR_014290, Thermo Fisher Scientific). Finally, the STR-based chimerism assessments were presented in tabulated form using Excel application for further analysis.

### Assessment of HMCC Phenotype by Flow Cytometry

The evaluation of the hematopoietic cell surface marker expression of T-cells (CD4), B-cells (CD19), and stem cells (CD45, CD90) was performed after fusion by flow cytometry (FC) (Gallios, Beckman Coulter, Brea, CA, USA). Moreover, the characterization of the selected hematopoietic cell population based on the cell surface markers expression was evaluated: T helper cells (CD3^+^/CD4^+^), T cytotoxic cells (CD3^+^/CD8^+^), T regulatory cells (CD4^+^/CD25^+^), hematopoietic cells (CD45^+^), NK cells (CD45^+^/CD56^+^), and primitive progenitor cells (CD34^+^/CD90^+^) at 7 days after the fusion by FC (Gallios, Beckman Coulter). The 1 × 10^6^ of parent UCB control cells and HMCC samples were suspended in the PBS staining buffer containing 1% BSA, and blocked with human BD Fc Block^™^ Reagent (BD Biosciences, Franklin Lakes, NJ, USA) for 5 min. Next, samples were incubated for 30 min on ice with the following anti-human monoclonal antibodies at saturating concentration: CD3 (APC, RRID:AB_314047, BioLegend, San Diego, CA, USA), CD4 (BD Pharmingen^™^ APC-Cy^TM^7, RRID:AB_398521, BD Biosciences), CD8 (BD Pharmingen^™^ Pacific Blue^™^, RRID:AB_397058, BD Biosciences), CD19 (APC/Cyanine7, RRID:AB_314248, BioLegend), CD25 (BD Horizon^™^ BV421, RRID:AB_2738555, BD Biosciences), CD34 (BD Pharmingen^™^ APC CD34, RRID:AB_398614, BD Biosciences), CD45 (Brilliant Violet 570^™^, RRID:AB_10899568, BioLegend), CD56 (BD Pharmingen^™^ APC, RRID:AB_398601, BD Biosciences), and CD90 (BD Horizon^™^ BV421, RRID:AB_2737651, BD Biosciences). After incubation, the samples were washed three times in a 1% BSA staining buffer, and analyzed using BD LSRFortessa^™^ Cell Analyzer (RRID:SCR_018655, BD Biosciences). Flowjo^™^ software (RRID:SCR_008520, Becton Dickinson) was used to determine the phenotype of the fused HMCC.

### Assessment of HMCC Propagation in Cell Culture

The UCB donor cells before fusion and the created HMCC after fusion were seeded into the low-adhesion culture T25 or T75 flasks at optimal density of 1 × 10^5^ cells/ml in an optimized medium (StemSpan™ Hematopoietic Cell Media, StemCell™ Technologies) with expansion supplement (H3000 + CD34^+^ supplement + 20% fetal bovine serum (FBS), StemCell™ Technologies) and cultured under standard conditions. The samples were analyzed on days 3, 7, and 10 using an inverted microscope (AE2000 inverted LED microscope, Catalog No. 01-258-030, Motic^™^, Kowloon City, Hong Kong) to assess propagation properties of the HMCC.

### Assessment of HMCC Viability by Flow Cytometry and Trypan Blue Staining

The FC analysis assessed HMCC viability using DAPI staining (n = 5). Briefly, a sample of 1 × 10^5^ of isolated unlabeled UCB, PKH26-, PKH67-, eFluor™ 670-labeled UCB control cells before fusion, and PKH26/PKH67/eFluor™ 670-labeled HMCC after fusion were stained with the DAPI Staining Solution (Miltenyi Biotec) at a final concentration of 0.1 μg/mL and directly analyzed by FC using the BD LSRFortessa™ Cell Analyzer (RRID:SCR_018655, BD Biosciences) and Flowjo^™^ (RRID:SCR_008520, Becton Dickinson) software. The viability of the UCB donor cells before cell fusion procedure and the viability of the created HMCC at 7 days after fusion procedure was determined with 0.4% Trypan Blue staining (Thermo Fisher Scientific), following the manufacturer’s instructions. The results were analyzed with an upright light microscopy (Leica DM4000 B, RRID:SCR_018895, Leica Microsystems) to determine the percentage of unstained cells and to evaluate the viability of UCB control cells before fusion and the created HMCC after cell fusion procedure.

### Assessment of HMCC Apoptosis by TUNEL Assay

To investigate apoptosis in the UCB donor cells and the created HMCC, TUNEL assay (Apo-BrdU™ TUNEL Assay Kit with Alexa Fluor™ 488 Anti-BrdU, A23210, Thermo Fisher Scientific) was performed at 7 days after cell fusion procedure (n = 3) and results were analyzed using FC (Gallios, Beckman Coulter). The APO™-BrdU TUNEL assay kit enables detection of the DNA strand breaks in the fused cells, which represent apoptosis. When DNA strands are cleaved by the nucleases, a large number of 3´hydroxyl ends are exposed. In the TUNEL assay, these ends are labeled with BrdUTP and terminal deoxynucleotidyl transferase. The BrdU incorporated into the DNA is then detected using a bright and photostable green-fluorescent Alexa Fluor488 dye-labeled anti-BrdU antibody. Both controls were performed according to the manufacturer’s instructions. The apoptotic positive control cells (DNase I treated) and negative control cells were prepared in the same way as HMCC samples. Briefly, cells were mixed with terminal deoxynucleotide transferase (TdT), BrdU, propidium iodide/RNase A staining buffer, and anti-BrdU antibody conjugated with AF488. The experiment was performed in triplicate. The AF488 fluorescence signal was detected with a flow cytometer (Gallios, Beckman Coulter) and analyzed with the Flowjo^™^ software (RRID:SCR_008520, Becton Dickinson). The TUNEL assay allowed to determine the level of apoptosis in the created HMCC.

### Assessment of HMCC Fusion Safety by Single Cell Gel Electrophoresis (COMET) Assay

To evaluate the genotype stability and possible DNA damage of the created and in vitro cultured HMCC, a single cell gel electrophoresis (SCGE) (COMET) assay (Abcam, Cambridge, United Kingdom) was performed at 7 days after cell fusion procedure (n = 2). The analyzed samples of HMCC were subjected to the alkaline lysis and further underwent a COMET assay assessment according to the manufacturer’s instructions (Cell Biolabs Inc., San Diego, CA, USA). After the COMET assay, the DNA was visualized using Vista Green fluorescent dye diluted in TE buffer (1:10,000), and analyzed under the fluorescent stereomicroscope (Leica MZ16FA, Leica Microsystems) equipped with a digital camera (QImaging^®^ Retiga-2000R Charge Coupled Device, QImaging). For the positive control, cells were treated with 100 μM of 3% hydrogen peroxide (H_2_O_2_) in order to induce DNA damage, whereas the propagated HMCC samples were left untreated. The experiment was performed in duplicate. The results were analyzed based on the presence of visible ‘comet’-like structures, which refer to the pattern of DNA damage that migrated through the electrophoresis gel.

### Assessment of HMCC Clonogenic Properties by Colony Forming Units Assay

To confirm maintenance of clonogenic properties, the Colony Forming Units Assa**y (**CFU) assay was performed on samples of the UCB donor cells and the created HMCC. A total of 1 × 10^3^ cells were seeded on an optimized methylcellulose-based medium (MethoCult^®^ H4034 Optimum, StemCell™ Technologies) in a 35 mm culture plate according to the manufacturer’s instructions and were further incubated for 14 days. After the CFU assay, slides were evaluated under the light microscope with the high objective lens (x50) (Leica DM 5500B Automated Upright Microscope, RRID:SCR_020219, Leica Microsystems) equipped with a digital camera (Leica DFC290 HD Color Digital FireWire Camera, Leica Microsystems), and colony number was presented in a chart for further analysis.

### Statistical Analysis

Statistical analysis was performed using Minitab software (RRID:SCR_014483, OriginLab Corp, Northampton, MA, USA). Assessments were performed in independent experiments with isolated unlabeled, PKH26-, PKH67-, and eFluor™ 670-labeled human UCB donor cells as the reference controls. Data are presented as mean ± standard deviation. Statistical differences between respective groups were assessed using two-tailed Student t-test or one-way Anova followed by Tukey’s post-hoc test. P values were considered significant below 0.05.

## Results

### Confirmation of Creation of New HMCC Line from Three Unrelated UCB Donors Via ex vivo PEG-mediated Cell Fusion

The study design of the ex vivo PEG-mediated cell fusion procedure of the human UCB cell derived from three unrelated donors (two males and one female) is presented on Fig. 1a. After the fusion procedure, the gating strategy used for HMCC sorting by FACS revealed 55.7% of ex vivo fused PKH26/PKH67/eFluor™ 670-labeled HMCC (Fig. 1b). The confirmation of the fusion efficacy and tri-chimeric state of the created HMCC from three unrelated UCB donor cells was proved by FC and CM (Fig. 1c-e). Firstly, prior to the fusion procedure, the UCB cells from each donor were separately labeled with either: PKH26 (red), PKH67 (green) or eFluor™ 670 (purple). The labeling of UCB cells assessed by FC confirmed the labeling efficacy for: PKH26 at 82.6%, for PKH67 at 86.5%, and for eFluor™ 670 at 98.6% (Fig. 1c). The fusion procedure efficacy, as confirmed by FC was 34.5%±14.7 (n = 5). The HMCC population presented > 95% purity as confirmed by FC (Fig. 1d). Following fusion procedure, the created HMCC were analyzed by CM (Fig. 1e). The UCB cells of male donor 1 were labeled with PKH26 (red), the UCB cells of female donor 2 labeled with PKH67 (green), the UCB cells of male donor 3 labeled with eFluor™ 670 (purple). The presence of the triple-labeled PKH26/PKH67/eFluor™ 670 cells of fuschia color (the merge of PKH26-red, PKH67-green, and eFluor™ 670-purple cell membrane dyes) confirmed creation of the new HMCC line. We confirmed the successful fusion of the human UCB cells from three unrelated donors and the creation of a new hematopoietic cell line of HMCC, confirmed by FC and CM analyses.

### Confirmation of Human Genotype Specific for Three Unrelated UCB Donors in the Created HMCC Line

**Fig. 2:**
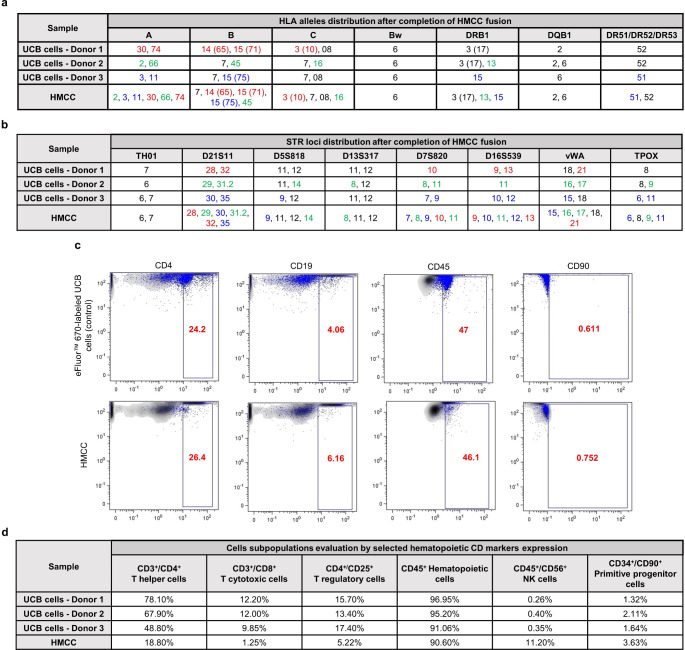
Confirmation of donor-specific genotype and maintenance of hematopoietic phenotype in the HMCC line created from three unrelated human UCB donors. (**a**) PCR-rSSOP analysis confirmed the presence of HLA class I and II antigens specific for each of the three unrelated UCB cells donors (donor 1 - red; donor 2 - green; donor 3 - blue) in the created HMCC after completion of the fusion procedure. (**b**) STR-PCR analysis confirmed chimerism in the created HMCC population by presence of all short tandem repeats (STR) loci in the DNA isolated from the HMCC population, derived from each of the three unrelated UCB cell donors (donor 1 - red; donor 2 - green; donor 3 - blue). (**c**) Representative dot-plots of hematopoietic and stem cell surface markers (CD4, CD19, CD45, CD90) expressed by: (upper row of dot-plots) eFluor™ 670-labeled UCB control cells, and (lower row of dot-plots) HMCC detected by FC after fusion. After fusion, the phenotype of the created HMCC is comparable with the phenotype of the parent UCB cells before fusion. (**d**) Flow cytometry assessment of the following hematopoietic cell subpopulations: T helper cells (CD3^+^/CD4^+^), T cytotoxic cells (CD3^+^/CD8^+^), T regulatory cells (CD4^+^/CD25^+^), hematopoietic cells (CD45^+^), NK cells (CD45^+^/CD56^+^), and primitive progenitor cells (CD34^+^/CD90^+^) assessed in the UCB donor cells and HMCC after 7 days of culturing. Increased expression of CD45^+^/CD56^+^ and CD34^+^/CD90^+^ markers was observed in HMCC at 7 days after fusion, when compared to the parent UCB donor control cells before fusion. There was a decrease in the number of the following cell subpopulations: CD3^+^/CD4^+^, CD3^+^/CD8^+^, CD4^+^/CD25^+^, and CD45^+^ in HMCC population when compared to the parent UCB donor cells before fusion

The PCR-rSSOP analysis of the HLA typing for class I and II antigens (A, B, C, Bw, DRB1, DQB1, and DR51/DR52/DR53) of the UCB donor cells before fusion and HMCC after fusion confirmed expression of the alleles specific for each of the three unrelated human UCB donors in the created HMCC (Fig. 2a). The STR-PCR analysis of the parent UCB cells before fusion and the HMCC cells after fusion confirmed the presence of all short tandem repeats (STR) loci (TH01, D21S11, D5S818, D13S317, D7S820, D16S539, vWA, and TPOX) specific for each of the three unrelated UCB donors in the HMCC line after fusion procedure (Fig. 2b). The presented data confirms the genotype specific for three unrelated UCB donors after ex vivo creation of the HMCC.

### Confirmation of Hematopoietic Phenotype of HMCC

To confirm that the fused HMCC are characterized by the hematopoietic phenotype specific for the three unrelated UCB donors, the FC analysis evaluated expression of the selected hematopoietic and stem cell surface markers (CD4, CD19, CD45, and CD90) in the created HMCC after cell fusion (Fig. 2c). The created HMCC expressed the following hematopoietic cell surface markers: 26.4% of CD4, 6.16% of CD19, 46.1% of CD45, and 0.752% of CD90. These values were comparable with the expression of hematopoietic markers in the parent UCB cells before fusion and revealed: 24.2% of CD4, 4.06% of CD19, 47% of CD45, and 0.611% of CD90 expression. The summarized data confirmed that the ex vivo cell fusion protocol does not alter the expression pattern of the selected hematopoietic markers of the created HMCC after fusion and is comparable with the surface markers of the parent UCB cells from three different, unrelated UCB donors. This confirms maintenance of the hematopoietic phenotype of the created HMCC. Evaluation of the CD markers expression in the selected subpopulations of the hematopoietic cells in the propagated HMCC revealed: 90.60% of CD45^+^ hematopoietic cells, 18.80% of CD3^+^/CD4^+^ T helper cells, 1.25% of T cytotoxic cells, 11.20% of CD45^+^/CD56^+^ NK cells, 5.22% of CD4^+^/CD25^+^ T regulatory cells and 3.36% of CD34^+^/CD90^+^ primitive progenitor cells (Fig. 2d). We confirmed increased expression of CD45^+^/CD56^+^ and CD34^+^/CD90^+^ markers, specific for the NK cells and primitive progenitor cells respectively, in the HMCC after fusion when compared to the parent UCB cells before fusion, confirming the stem-like and regenerative potential of the fused HMCC.

### Confirmation of Viability and Low Apoptosis Level of HMCC Line

**Fig. 3:**
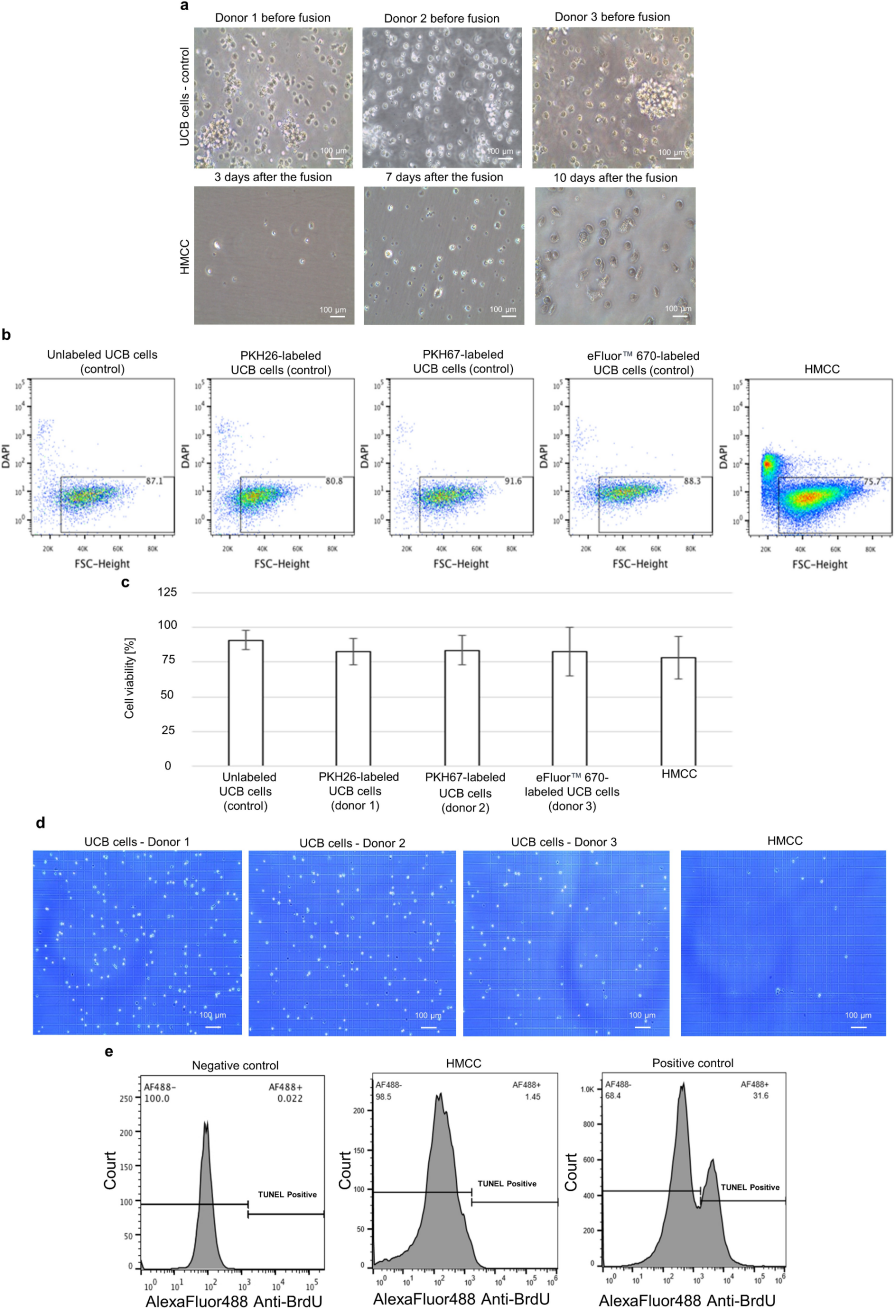
Confirmation of HMCC propagation, viability, and low apoptosis level in the UCB donor cells and in the created HMCC. (**a**) Representative images of: (upper row panels) human UCB cells from three unrelated donors before fusion procedure and (lower row panels) HMCC during cell propagation at 3, 7, and 10 days of culture after fusion. Images from cell cultures captured under an inverted microscope (AE2000 inverted LED microscope), scale bar: 100 μm. (**b-c**) Qualitative and quantitative analysis of HMCC’s viability after sorting using DAPI staining: (**b**) Representative flow cytometry dot-plots presenting DAPI staining vs. FSC-height of UCB control cells and HMCC population after fusion procedure (n = 5) (in sequential order from the left): isolated UCB cells, PKH26-labeled UCB control cells, PKH67-labeled UCB control cells, eFluor™ 670-labeled UCB control cells, and PKH26/PKH67/eFluor™ 670-labeled HMCC after fusion; gate indicates the viable DAPI^-^ cells; (**c**) Comparison of the number of viable cells based on flow cytometry assessment of DAPI staining of UCB control cells before fusion and HMCC population after fusion procedure (in sequential order from the left): isolated UCB cells, PKH26-labeled UCB control cells, PKH67-labeled UCB control cells, eFluor™ 670-labeled UCB control cells, and PKH26/PKH67/eFluor™ 670-labeled HMCC after fusion. No statistically significant difference in cell viability was observed between UBC control cells prior to fusion and HMCC post-fusion. (**d**) Assessment of HMCC’s viability by Trypan Blue staining at 7 days after fusion (in sequential order from the left): first three images, the viability of parent UCB cells from three unrelated donors and the last image on the right, the fused HMCC, confirming the viability maintenance of HMCC after fusion procedure. Images were captured using an upright light microscope (Leica DM4000 B), scale bar: 100 μm. (**e**) Detection of apoptosis in the fused HMCC assessed by TUNEL assay at 7 days after fusion (n = 3), revealed (in sequential order from the left): negative control (0.022% of apoptotic cells), HMCC (1.45% of apoptotic cells), and positive control (31.6% of apoptotic cells), revealing low level of apoptosis in the created HMCC line, further confirming safety of in vitro propagation of HMCC after fusion procedure

The images of the in vitro cultured HMCC were obtained at 3, 7, and 10 days after cell fusion and were compared to the images of the cultured human UCB donor control cells. The created HMCC after in vitro expansion presented a regular morphology of hematopoietic cells and a high proliferation rate when seeded at optimal density of 1 × 10^5^ cells/ml. (Fig. 3a). The FC analysis of cell viability at each stage of the fusion procedure using DAPI staining indicated that there was no statistically significant difference in cell viability (> 75%) observed between unlabeled and fluorescently labeled UCB control cells prior to fusion and HMCC post-fusion (p > 0.05, Fig. 3b-c). There was no statistical difference in the average viability of HMCC (62.2%±23.82) and UCB donor control cells (average viability of UCB donor 1: 96.5%±3.96, UCB donor 2: 94.4%±4.94, vs. UCB donor 3: 92.5%±3.4, p > 0.05) after 7-day culture as assessed by Trypan Blue staining (Fig. 3d). Further, the evaluation of apoptosis level in the HMCC population as performed by the TUNEL assay, showed a low level of 1.45% anti-BRDU-positive cells in the HMCC population at 7 days after fusion procedure (Fig. 3e). This data confirmed the maintenance of HMCC viability following fusion and in vitro culturing, and therefore supports the HMCC’s potential for in vivo survival.

### Confirmation of Safety and DNA Stability of HMCC Line

**Fig. 4:**
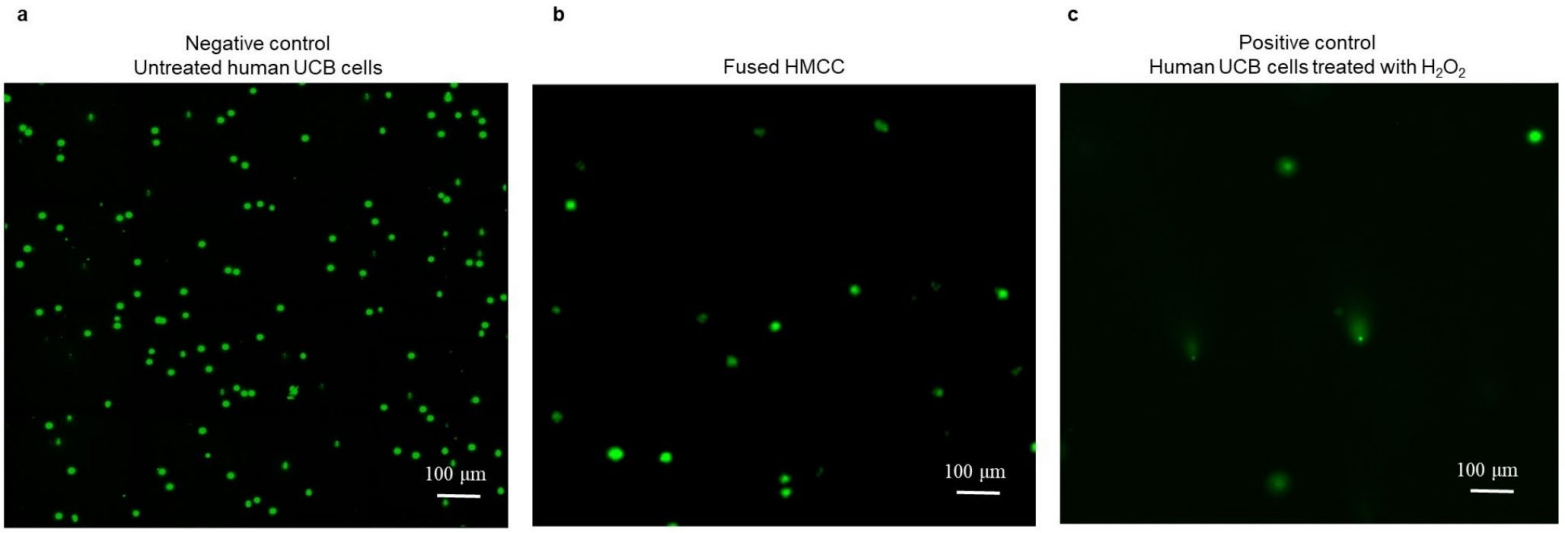
Confirmation of fusion safety and DNA stability of HMCC created by fusion of UCB cells from three unrelated human donors assessed by single cell gel electrophoresis (SCGE) COMET assay. (**a-c**) Representative fluorescent images of COMET assay at 7 days after fusion (n = 2) of: (**a**) Negative control of untreated human UCB cells; (**b**) Fused HMCC and the absence of the characteristic ‘comet’-like structures, confirming the DNA stability in the created HMCC; and (**c**) Positive control of human UCB cells treated with H_2_O_2,_ presenting H_2_O_2_-induced DNA damage, visible as a ‘comet’-like structure. Green: Vista Green DNA Staining Solution (nucleus stain). Images were captured using a fluorescence stereomicroscope (Leica MZ16FA), scale bar: 100 μm

In order to evaluate the possible DNA damage in the in vitro cultured HMCC, a single cell gel electrophoresis (COMET) assay was performed (Fig. 4) at 7 days after fusion procedure. Analysis of the images visualized under the microscope proved that there were no ‘comet’-like structures in the UBC negative control cells (Fig. 4a) and in the fused HMCC (Fig. 4b), confirming the absence of DNA damage, whereas the ‘comet’-like structures were observed in the positive control cells treated with 100 μM of 3% H_2_O_2_ used to induce DNA damage (Fig. 4c). Therefore the genetic material of the HMCC was not determined as damaged, further confirming the safety of the ex vivo PEG-mediated fusion procedure and the DNA stability of the created HMCC.

### Confirmation of Clonogenic and Proliferative Properties of HMCC Line

**Fig. 5:**
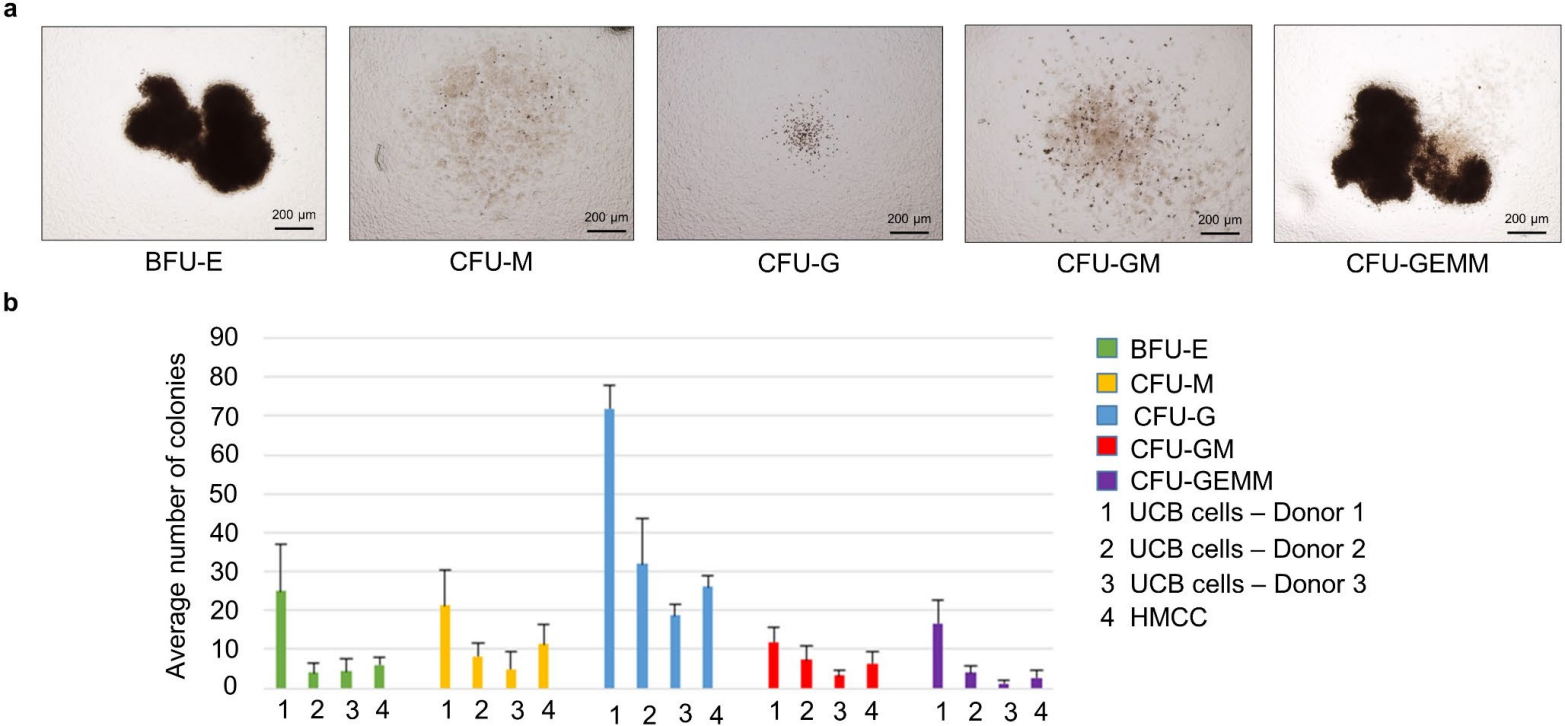
Confirmation of clonogenic properties of the created HMCC by CFU assay. (**a**) Representative images of (in sequential order from left): burst-forming unit - erythroid (BFU-E); macrophage colony forming unit (CFU-M); granulocyte colony forming unit (CFU-G); granulocyte macrophage colony forming unit (CFU-GM); and granulocyte erythroid macrophage megakaryocyte colony forming unit (CFU-GEMM), taken from cell culture under a light microscope (Leica DM 5500B Automated Upright Microscope), scale bar: 200 μm. (**b**) Comparative analysis of the average number of colonies (BFU-E, CFU-M, CFU-G, CFU-GM, and CFU-GEMM) created by each of the UCB cell donors before cell fusion and HMCC after cell fusion. The average number of colonies of the created HMCC did not differ from the average number of colonies derived from three (donor 1–3) UCB cell donors

The CFU assay confirmed the maintenance of clonogenic properties of the ex vivo created HMCC following the fusion procedure. Representative images of the created colonies show that HMCC have potential to differentiate into all tested classes of myeloid and erythroid progenitor-derived cells, including: erythroid burst-forming units (BFU-E), macrophage colonies (CFU-M), granulocyte colonies (CFU-G), granulocyte macrophage colonies (CFU-GM), and granulocyte erythroid macrophage megakaryocyte colonies (CFU-GEMM) (Fig. 5a). There was no statistical difference between the average number of CFU colonies (BFU-E, CFU-M, CFU-G, CFU-GM, and CFU-GEMM) between the sorted HMCC and each of UCB donor control cells (Fig. 5b). These results additionally confirmed the safety of the HMCC for in vivo application as a therapy by showing the lack of uncontrolled HMCC proliferation.

## Discussion

The challenges associated with transplant rejection have sparked significant interest in medical research. As the number of solid organs and bone marrow transplants performed annually in the United States continues to rise, there is a pressing need for new therapeutic approaches to modulate the immune response [1]. Currently, the reliance on lifelong IS therapy to prevent transplant rejection leads to severe side effects that negatively impact the lifespan of transplant recipients [2, 3]. Therefore, the introduction of accessible and effective therapeutic strategies for tolerance induction in solid organs, bone marrow, and VCA is crucial.

Cellular therapies, particularly those applied in BMT protocols, have shown promise in modulating the immune response and eliminating the need for lifelong multidrug IS. These therapies facilitate the development of mixed chimerism, which allows for the induction and maintenance of tolerance. Our previous work introduced the concept of donor-recipient chimeric cells (DRCC) therapy, derived from bone marrow cells of VCA transplant donor and recipient, which demonstrated promising results in establishing a clinically relevant ex vivo PEG-mediated cell fusion protocol and the creation and characterization of chimeric cell line in a preclinical experimental rodent model. This personalized donor-recipient cellular therapy significantly prolonged the survival of fully MHC-mismatched VCA transplants by inducing long-term mixed chimerism [6, 13].

Building upon the success observed in the VCA rat model, the next step towards clinical application involved the development of a new therapy based on ex vivo fusion of human bone marrow-derived CD34^+^ cells derived from two unrelated donors, resulting in the creation of a new cell line of human hematopoietic chimeric cells (HHCC) [16]. Through in vitro characterization, we successfully assessed the viability, phenotype, polyploidy, genotype, clonogenic, tolerogenic, and immunomodulatory properties of HHCC therapy for tolerance induction in solid organ, bone marrow, and VCA transplantation. The success of HHCC therapy based on bone marrow-derived cells has encouraged us to explore new donor cell candidates as alternative treatment options in transplantation.

UCB has emerged as a reliable option for tolerance induction in SOT, BMT, and VCA. It is also a valuable treatment option for hematological malignancies. In patients with acute leukemia who lack a suitable 8/8 HLA-matched BM donor, UCB serves as an alternative source of hematopoietic stem cells for transplantation. In pediatric cases, the recurrence rate of leukemia after a 4/6 HLA-matched UCB transplant is lower than after an 8/8 HLA-matched BM transplant [21]. Additionally, the treatment failure rate is similar between a 4/6 HLA-matched UCB transplant and a 5/6 HLA-matched UCB transplant when compared to an 8/8 HLA-matched BM transplant [21]. The choice of UCB cell dose has an impact on patient outcomes, with higher engraftment observed after a 5/6 HLA-matched UCB transplant with a high cell dose compared to a lower cell dose [22, 23]. In the adult population, there is no statistical difference in leukemia recurrence rate between patients who received 4–5/6 HLA-matched UCB transplants or 7–8/8 HLA-matched BM transplants [24]. Furthermore, the incidence of acute and chronic GVHD is lower after 4–6/6 HLA-matched UCB transplants compared to 7/8 HLA-matched BM transplants. Rates of acute and chronic GVHD are similar between patients who received 4–6/6 HLA-matched UCB transplants and those who received 8/8 HLA-matched BM transplants [24–27]. Consequently, UCB transplants require a lower level of HLA-matching compared to BM transplants while yielding similar outcomes, making UCB transplants an effective alternative in the absence of 8/8 HLA-matched BM donors.

Based on the literature reports on the successful use of UCB transplants in patients with hematologic diseases, including leukemia, our Microsurgery Laboratory selected UCB cells as the potential candidate for the development of a novel chimeric cell therapy as an alternative approach to BM-based cellular therapy. Initially, we confirmed the creation of a human umbilical di-chimeric (HUDC) cell line by ex vivo PEG-mediated fusion of UCB cells from two unrelated human donors. The in vitro characterization of HUDC cells encompassed their genotype, phenotype, viability, safety, and clonogenic properties. This innovative approach allowed us to achieve a closer donor-specific HLA-match as a first step towards creating chimeric cell lines which could be comparable to a related HLA-matched donor.

Building upon the success of HUDC cell therapy, we proceeded to develop the next generation of multi-chimeric cells with an even closer and universal HLA-match. This led to the creation of HMCC therapy, obtained by the fusion of UCB cells from three unrelated human donors. Compared to di-chimeric HUDC cells representing two HLA-matches, the created HMCC represented tri-chimeric cells with three HLA-matches, thus increasing a chance of a closer match to the recipient. Similar to HUDC cells, we confirmed the feasibility and safety of the ex vivo PEG-mediated fusion protocol for creating HMCC. Additionally, we characterized the phenotype, genotype, viability, safety, and clonogenic properties of the fused HMCC. The fusion of genetic material from three unrelated UCB donor cells in HMCC was confirmed by PCR-rSSOP and STR-PCR analyses which revealed the presence of HLA class I and II antigens, as well as STR loci specific to each of the parent UCB donor cells. This analysis effectively validated the application of the ex vivo fusion procedure for the creation of multi-chimeric cell lines as confirmed in this study by fusion of UCB cells from three unrelated human donors. Phenotype analysis demonstrated the expression of hematopoietic and stem cell surface markers in propagated HMCC, further validating the hematopoietic origin and maintenance of the hematopoietic phenotype of HMCC after fusion procedure. Moreover, the viability of HMCC was maintained and was comparable to the viability of UCB donor cells for up to 7 days of culture following the fusion procedure. This indicates that the ex vivo PEG-mediated fusion protocol does not adversely affect the viability of the created HMCC. Additionally, the TUNEL assay revealed a low level of apoptosis in HMCC, further confirming the stability of the created HMCC line. To ensure the safety of the ex vivo fusion protocol and assess the long-term DNA stability of HMCC, the COMET assay was conducted on the UCB donor cells before fusion and on the created HMCC after fusion. The absence of ‘comet’-like structures, which indicate DNA damage, in the created HMCC demonstrated the lack of DNA damage after fusion. In order to consider the potential clinical application of HMCC therapy, we examined the clonogenic properties of the created HMCC. The characterization confirmed the differentiation potential of HMCC into granulocyte, erythroid, macrophage, and megakaryocyte progenitor cells after 14 days of cell culture. This differentiation capacity was comparable to that of the human UCB donor cells. The formation of colonies in the cell culture further supported the proliferative properties of the created HMCC.

The mechanism of action of the created HMCC relies on the genetic match achieved between the donors and recipient after ex vivo PEG-mediated fusion procedure of human UCB cells derived from three unrelated UCB donors. As a result, the created trimeras carry HLA class I and class II alleles, and STR loci, originating from each of the UCB donors. This confirmation is established through lymphocytotoxicity test and PCR-STR analysis, respectively. Thus, the generated HMCC line represents a matching of HLA profiles from the three donors with the recipient’s HLA, highlighting a novel approach to achieve a closer HLA-match between the donors and the recipient. As a consequence, it is anticipated that HMCC will be better tolerated by the recipient, mitigating the likelihood of triggering a significant immune response. Therefore, this approach holds the potential to revolutionize the compatibility between donors and recipients, offering a substantial advancement in the realm of transplantation medicine.

In summary, in this study we have successfully created a new line of HMCC through ex vivo PEG-mediated fusion of human UCB cells from three unrelated donors. The tri-chimeric state of HMCC was confirmed through various analyses. The hematopoietic phenotype of HMCC closely resembled that of UCB donor cells, indicating the preservation of functional properties of the created HMCC. The maintained HMCC viability, low level of apoptosis, and the absence of genotoxic effects, suggest HMCC safety for therapeutic use.

The successful creation of hematopoietic cell lines, including HUDC cells and HMCC, from human UCB donor cells highlights the potential of UCB-based chimeric cell therapy as a promising “off-the-shelf” approach for achieving a closer donor-specific HLA-match in tolerance induction for transplant medicine and regenerative medicine.

Building upon the promising results of the in vitro characterization of the created HMCC line, as summarized in this study, our aim is to advance the HMCC therapy towards clinical trials while exploring its potential application in transplantation. In pursuit of this goal, we plan to further assess and validate the safety, migratory pathways, and overall efficacy of the HMCC therapy using an in vivo murine VCA model. To achieve this purpose, we will employ the preclinical NSG mouse model, which is widely recognized and established in transplantation research. This in vivo study will provide valuable insights into the clinical applicability of HMCC therapy and further enhance our understanding of its potential benefits in the field of transplantation.

Based on the preclinical in vivo studies confirming safety and efficacy of HMCC, in the clinical scenario, the manufacturing of the multi-chimeric cells will take place in the Cell Bank facilities under Good Manufacturing Practice (GMP) conditions. This will allow for the regulatory approvals of HMCC in preparation for the Phase 1 clinical trials.

To the best of our knowledge, this study reports the first successful creation of a novel human hematopoietic cell line of HMCC through ex vivo PEG-mediated fusion of UCB cells derived from three unrelated donors. The capacity to selectively match the recipient with three unrelated donors presents a significant advantage in inducing and maintaining tolerance in SOT, BMT, and VCA, thereby addressing the persistent challenge of donor shortage and improving transplant patient outcomes. In addition, HMCC therapy provides the potential for tailored personalized treatment with a closer donor-recipient HLA-match compared to HUDC cell therapy.

## Conclusions

This study demonstrated the successful creation of a novel HMCC line derived from three unrelated human UCB donors. The characterization of HMCC genotype, phenotype, viability, safety, and clonogenic properties, confirmed the feasibility and safety of HMCC for therapeutic applications. HMCC therapy offers a new cellular therapeutic approach for achieving mixed chimerism and tolerance induction in the fields of SOT, BMT, and VCA. This novel therapeutic strategy holds promise for improving patient outcomes and addressing persistent challenges associated with transplantation.

### Electronic Supplementary Material

Below is the link to the electronic supplementary material.

Supplementary Material 1

## Data Availability

All data generated or analyzed during this study are included in this published article and are available for presentation upon request.
