# Supplementary material for: Human Multi-Chimeric Cell (HMCC) Therapy as a Novel Approach for Tolerance Induction in Transplantation
Source: Stem Cell Rev Rep. 2023 Aug 21;19(8):2741–55. doi: 10.1007/s12015-023-10608-z (PMC10661767; doi:10.1007/s12015-023-10608-z)
Supplement: Supplementary file 1 — Supplementary Material 1 [file 12015_2023_10608_MOESM1_ESM.pdf]

July 28, 2023

Mariusz Ratajczak, M.D., Ph.D., D.Sc.
Editor-in-Chief
Stem Cell Reviews and Reports
University of Louisville
Louisville, KY, USA

RE: *"**Human Multi-Chimeric Cell (HMCC) Therapy as a Novel Approach for Tolerance Induction in Transplantation**"*

Dear Professor Ratajczak,

Enclosed please find our original manuscript entitled: *"Human Multi-Chimeric Cell (HMCC) Therapy as a Novel Approach for Tolerance Induction in Transplantation"* for your consideration of publication in the *Stem Cell Reviews and Reports* journal.

We confirm that this manuscript or any parts of its content has not been previously published or are currently under consideration or are published in another journal and will not be sent to another journal until a decision regarding publication is made by the *Stem Cell Reviews and Reports* journal.

In this article, we proposed a novel cellular therapy of *Human Multi-Chimeric Cell (HMCC)* created via *ex vivo* PEG-mediated fusion of Umbilical Cord Blood (UCB) cells from three unrelated human donors. We propose this new hematopoietic cell line of HMCC as an alternative approach to bone marrow (BM)-based therapies in support of bone marrow, solid organ, and vascularized composite allotransplantation.

This study confirmed the feasibility of creation and the chimeric state of HMCC by flow cytometry, and confocal microscopy. Moreover, we characterized *in vitro* the genotype, hematopoietic phenotype, viability, safety, and clonogenic properties of the created HMCC line.

As the preferred Reviewers for this manuscript, we would like to recommend the following experts in the field of regenerative medicine and cell-based therapies:

1. Mariusz Ratajczak MD, PhD, DSc - mariusz.ratajczak@louisville.edu
2. Safak Uygur MD - safakuygur@gmail.com
3. Yalcin Kulahci MD - yakulahci@yahoo.com

We would like to request excluding the following Reviewers due to the competitive conflict of interest:

1. Józef Dulak PhD - jozef.dulak@uj.edu.pl
2. Jerzy Kupiec-Weglinski MD, PhD - jkupiec@mednet.ucla.edu
3. Maciej Kurpisz MD, PhD - maciej.kurpisz@igcz.poznan.pl

All authors approved the manuscript, agreed with its submission to the *Stem Cell Reviews and Reports* journal, and had no conflicts of interest to disclose.

Below is the Authors list :

1. Maria Siemionow MD, PhD, DSc - siemiom@uic.edu *https://orcid.org/0000-0001-6372-6122*
2. Joanna Cwykiel PhD - cwykiel@gmail.com *https://orcid.org/0000-0002-5784-1156*
3. Sonia Brodowska BSc - soniaa.bro@gmail.com *https://orcid.org/0000-0002-0546-0060*
4. Lucile Chambily BSc, MD - lucile.chambily@gmail.com *https://orcid.org/0009-0008-3377-3284*

I hope that this study, introducing HMCC line as a novel therapeutic approach for bone marrow, solid organ, and vascularized composite allotransplantation, will be of interest to the readership of *Stem Cell Reviews and Reports* journal.

Thank you for your consideration.

Sincerely,

Maria Siemionow, MD, PhD, DSc
Professor of Orthopaedics
Director of Microsurgery Research
University of Illinois at Chicago, Chicago, IL, USA
Phone: 312-996-2578, Fax: 312-996-8789
Correspondence to: siemiom@uic.edu
